# 

**Published:** 1895-08

**Authors:** 


### CONTENTS.

ORIGINAL ARTICLES—

A Case of Multiple Neuritis. By H. F. Harris, M. D., Atlanta... 393
The Nature and Treatment of Acne. By Bernard Wolff, M. D., Atlanta... 400
Report of a Case of Triplets. By T. W. Basinger, Petersburg, Indiana... 407
Palmetto Wine. By Ira W. Porter, M. D., Mobile, Ala... 408
A Case of Facial Neuralgia Cured by the Removal of a Tooth Containing Pulp Calculi. By D. S. Arnold, M. D., Atlanta.. 409
New York Letter... 410

SOCIETY REPORTS—

Richmond Academy of Medicine and Surgery... 413

SELECTIONS AND ABSTRACTS—

Some New Theories of Digestion .......... 417
Clitoridectomy .......... 420
Puerpueral Sepsis .......... 421
Pruritus Ani .......... 422
The Immunity Conferred by an Attack of Infectious Disease .... 423
Medicine in Fiction .......... 424
Collodion in Plastic Operations .......... 425
The Warm Bath in Insomnia .......... 426
Ichthyol in Erysipelas .......... 427
Chloride of Lime in Snake Bite .......... 427

EDITORIAL—

Bicycling for Women .......... 429
Notes .......... 433
Book Reviews .......... 435
Special Notes .......... 436
Prescriptions .......... 440

